# Misleading Elevation of Troponin T caused by Polymyositis

**Published:** 2013-06

**Authors:** Teena Dhir, Ning Jiang

**Affiliations:** 1 14511 Pine Court, Riverside, CA, 92503, USA;; 2 Prince George’s Hospital Center, 3001 Hospital Drive, Cheverly, MD, 20785, USA

**Keywords:** false positive Troponin T, Polymyositis, Troponin T, Troponin I

## Abstract

**Background:**

Elevations of cardiac enzymes are commonly used to indicate myocardial ischemia, but they can be elevated due to other conditions. Different forms of Troponin (cTnT, sTnT, cTnI), can cause cross-reactivity in the Troponin T assay, leading to false positives. This report describes a patient with polymyositis who had elevated Troponin T, but no cardiac abnormalities. The purpose is to show that Troponin T, which is believed to be solely from cardiac muscle breakdown, can be seen in inflammatory muscle disease, so Troponin I should be used instead.

**Description:**

This is a case report of a 70-year-old woman with a history of diabetes, hypertension, gout and polymyositis, who presented with one-day history of lightheadedness and abdominal pain. To rule out myocardial ischemia, cardiac enzyme testing was ordered which showed elevated CK, CK-MB, and Troponin T. A full cardiac workup was performed which showed no signs of ischemia. Troponin I was <0.05 ng/mL, (normal).

**Discussion:**

In inflammatory myositis, there are elevations in many cardiac markers due to non-cardiac causes, which could be related to muscle regeneration and gene expression. This is not seen certain isoforms of Troponin I, specifically cardiac Troponin I.

**Conclusion:**

In patients with history of diabetes and other comorbidities, silent myocardial ischemias should be ruled out. Non-cardiac elevations in Troponin T can be seen in patients with inflammatory, so Troponin I should be ordered to get an accurate interpretation. Patients with inflammatory myopathies can have elevations in CK, CK-MB, and Troponin T, but not Troponin I.

## INTRODUCTION

Currently, elevations in cardiac enzymes like creatine kinase- cardiac muscle (CK-MB) and Troponin T are commonly used as indications of myocardial ischemia, but these enzymes can be falsely elevated (meaning they are elevated, but not due to a cardiac cause) in many other pathophysiological constellations as well. Troponin T is considered to be a highly sensitive marker of myocardial injury, but it can be falsely elevated in conditions like renal failure, subarachnoid hemorrhage, myasthenia gravis, rhabdomyolysis, rheumatoid arthritis and inflammatory muscle diseases like dermatomyositis and polymyositis (1, 6). In patients with myositis, an accurate cardiac workup is needed because myocardial ischemia is still a major cause of death in patients with dermatomyositis and polymyositis (3). Patients with myositis have a 16-fold increased risk of myocardial ischemia than the normal population. The risk was found to be higher in females than in males as well (3). A matter of particular interest is the differentiation between a true elevation in cardiac enzymes in these patients, and a false positive. In our case report a patient with polymyositis presented to the emergency department (ED), complaining of right sided abdominal pain and was found to have abnormal cardiac enzymes with raised Troponin T levels.

## CASE REPORT

**Figure 1:**
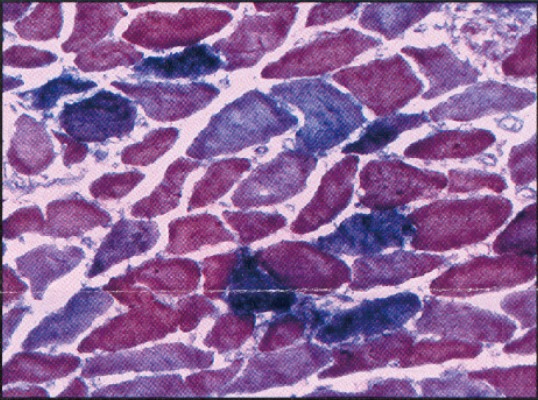
Deltoid muscle biopsy from patient with frozen SDH/COX stain showing COX negative fibers with inflammatory myopathy-diagnosis: polymyositis/ cytochrome oxidase overlap syndrome.

A 70 year old woman with history of diabetes type 2, hypertension, gout and polymyositis was admitted to our hospital. She presented to the ED with symptoms of light-headedness, dizziness and pre-syncopal episode earlier in the morning, along with a sudden onset of generalized body weakness. She denied any loss of consciousness or palpitations before presentation to the ED. She also complained of sharp pain in the upper right abdominal quadrant, verbally stated as 10/10 in pain, non-radiating, for 4 days duration, with associated nausea. Patient recently had right kidney surgery with a nephrostomy tube draining 5-10 ml of blood as well for the past 4 days. She had past history of nephrolithiasis one year ago, leading to severe right-sided hydronephrosis (Figure 1).

Home medications were as follows: Cymbalta, 60 mg, oral, daily; Nexium 40 mg, oral, daily; Acetaminophen with Tramadol, 325 mg, oral, every 6 hours as needed; Glipizide 2.5 mg, oral, daily; Amlodipine 10 mg, oral, daily; Phenazopyridine 200 mg, oral, three times a day; Indomethacin 50 mg, oral, daily; Enablex 15 mg, PO, daily, and Methotrexate 10 mg, oral, weekly. The patient has a history of hypertension for 50 years, type II diabetes for 2 years and polymyositis for the past 5 years. Patient had no family history of cardiovascular disease, heart attack or stroke.

Admission vitals: Temperature 97.8°F, pulse rate of 80 beats-per-minute, blood pressure of 87/61 mmHg which improved to 130/83 mmHg after receiving intravenous fluids, oxygen saturation of 98% on room air. Physical exam: patient was awake and alert, with only pertinent findings of right upper quadrant tenderness. Neurological exam showed normal deep tendon reflexes, muscle strength was 3/5 bilaterally in the legs and 4/5 bilaterally in the arms, with a strong grip. The remainder of the physical exam was normal. Laboratory findings at admission: complete blood count, chemistry panel, magnesium, phosphorus, and lactate levels all within normal limits. Urine analysis showed moderate bacteria, significant proteinuria with nitrates and leukocytes present. Patient had glycosylated hemoglobin A1c of 6.4.

After admission to the internal medicine unit, the patient was examined to rule out causes of sepsis including any type of upper or lower urinary tract infection that could lead to the patient’s presentation. Blood and urine cultures were obtained which showed no growth at 3 days. Computer Tomography (CT) of the abdomen showed a small right kidney with reduced cortex thickness and with small amount of perinephric fluid accumulation with no signs of hydronephrosis. Patient was started on Imipenem to cover for pyelonephritis, and the right upper quadrant pain resolved in 4 days with stable vital signs.

To assess if the symptoms of lightheadedness, sudden generalized weakness and pre-syncopal episode were due to a cardiac cause (e.g. silent myocardial ischemia) or transient ischemic attack, cardiac evaluation, along with doppler ultrasound of the carotid arteries and CT of the brain, were performed. CT showed no signs of ischemia or hemorrhage, and an electrocardiogram (ECG) from the day of admission showed left axis deviation and signs of possible old anterior infarction with associated ST-T/ T wave abnormalities, unchanged compared to the ECG from a year ago. Carotid doppler was normal with no signs of stenosis. Cardiac enzymes results are shown in Table 1.

**Table 1:** Comparison of creatine kinase (CK), creatine kinase- cardiac muscle (CK-MB) and Troponin T showing no changes during hospital stay from baseline

Date (after admission)	CK (in U/L)	CK-MB (in ng/mL)	Troponin T (in ng/mL)	CK-MB/Total CK (in %)

**Day 1**	524	10.0	0.148	1.9
**Day 2**	438	7.4	0.153	1.7
**Day 3**	406	7.5	0.131	1.8
**Day 4**	358	6.7	0.138	1.9
**Day 5**	463	8.8	0.162	1.9
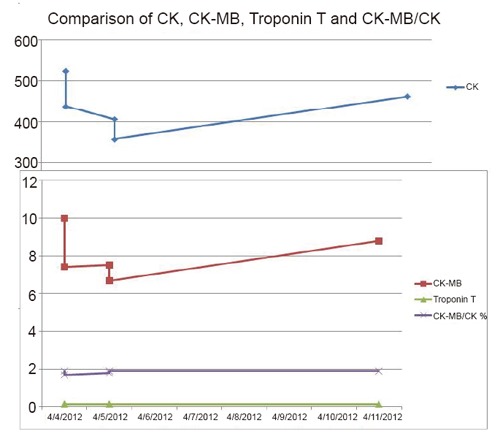				

Normal reference values (may change depending on provider): CK: 38-176 U/L; CK-MB: 0-3 ng/mL; Troponin T: <0.01 ng/mL; CK-BM/Total CK: <1%.

From patient’s admission one year ago, CK was 484 U/L, and CK-MB was 12.3 mg/mL, indicating elevated CK and CK-MB were due to polymyositis muscle breakdown. Further laboratory findings showed myoglobin 186.2 ng/mL (normal= 0-85 ng/mL), haptoglobin 337 mg/dL (normal= 41-165 mg/dL), with normal blood uria nitrogen (BUN), creatinine and thyroid function. Though there were no acute changes on ECG, the patient is at increased risk of coronary artery disease with a stress test pretest probability of 10%-90% risk due to her age and history of diabetes and hypertension. To further assess causes of the abnormal ECG, 2D echocardiogram of the heart was obtained which showed left ventricle ejection fraction of 65% to 70% with normal atria and ventricles. There was mild mitral regurgitation and mild tricuspid regurgitation with right ventricle systolic pressure of 43 mm, which was consistent with mild pulmonary hypertension. To ensure there were no signs of myocardial ischemia a nuclear stress test was performed instead of the traditional stress test due to the patient’s muscle weakness. The nuclear stress test measures blood flow to the myocardium at rest and during exercise, simulated with the use of medications. The study showed normal myocardial perfusion with normal left ventricular centrality at rest, and an ejection fraction of 54%, all within normal limits. Though all the tests performed indicated there were no signs of myocardial ischemia, we decided to obtain a Troponin I level, which was <0.05 ng/mL (normal 0.00-0.14 ng/mL). By performing all these tests we had concluded that there was no cardiac cause for the increased CK-MB and Troponin T, reflecting that patient’s Troponin T elevations must have been due to polymyositis.

## DISCUSSION

Polymyositis is an inflammatory muscle disorder caused by auto-antibodies against skeletal myositis, which results in proximal muscle weakness and muscle disorder. Treatment for this systemic disease requires the use of immune-suppressants such as corticosteroids, azathioprine or methotrexate, but other organs can also be affected by this disease. Patients with inflammatory myositis are at increased risk of cardiovascular complications including arrhythmias, myocardial infarction and myocarditis because of antibody cross-reactivity with the cardiac muscle (3). The most common cardiac complication in this population is congestive heart failure, but most patients have subclinical symptoms with subtle ECG changes like heart blocks, PR interval prolongation, Q waves and nonspecific ST-T wave changes. The most common ECG changes seen in patients with polymyositis are left anterior hemiblock and right bundle branch block (3).

As seen in this case report, in patients with polymyositis CK and CK-MB are usually elevated due to the constant muscle breakdown and rebuilding secondary to immune mediated destruction of skeletal muscle. CK is an enzyme used to carry and release energy, and is found in various tissues in the body. It has 3 different isoenzymes, CK-BB (brain), CK-MM (skeletal muscle), and CK-MB (cardiac muscle). As the skeletal muscle begins to regenerate, it has been shown that there is an increase in CK-MB in the new skeletal muscles due to re-expression of embryonic genes, and this increase in CK-MB can represent anywhere from 5-10% of total CK (9, 10), as seen in this patient.

Based on the impairment and regeneration of skeletal muscle, this tissue also releases Troponin T. Troponin as a regulatory protein is responsible to stabilize and activate actin for muscle contraction, and is composed of three subunits: Troponin T (TnT), Troponin I (TnI), and Troponin C (TnC). Troponin T binds to tropomyosin to generate a complex, Troponin I binds to actin to block its active site, and Troponin C binds to calcium and induces enzyme activation. Skeletal muscle shows specific Troponin groups (sTnT, sTnI, sTnC), which are released in case of alterations focusing on skeletal muscle; cardiac forms of Troponin T (cTnT), Troponin I (cTnI) and Troponin C (cTnC) also exist, which are released when myocardial damage occurs (10). As the skeletal muscle regenerates after damage, it has been shown that the muscle reverts back to expressing its embryonic forms of CK-MB, as well as cardiac Troponin T (cTnT) but no cTnI elevation could be seen (10). These laboratory findings provoke incorrect clinical interpretation in patients with a suspicion of myocardial damage, showing elevations of Troponin T and CK-MB to indicate that there is possible damage to the myocardium (10), and in patients with polymyositis this elevation can point at false positives, or even mask a true event of myocardial injury. Recently, improvements in the specificity of the Troponin T assay are available, but cross-reactivity between sTnT and cTnT is still present and, in patients with inflammatory myositis, elevations in Troponin T (cTnT) have been seen (5).

In studies comparing sTnI elevations and cTnI elevations in patients with inflammatory myositis, it has been shown that there is a significant correlation in CK and CK-MB elevations based on repeated skeletal muscle regeneration (4, 7). There is a strong correlation between elevations in CK and cTnT, as well as CK-MB and cTnT (7). It has also been shown that there is a correlation between sTnI elevations with CK-MB, but *no correlation* between cTnI and CK-MB (4, 7) indicating that cTnI was not expressed in regenerating skeletal muscles (10). In the past, CK-MB to total CK ratio of greater than 3 was used as an indicator that there is myocardial damage; but in patients with active myositis the CK-MB to total CK ratio can also provoke a false positive finding. This problem could be circumvented by examining cTnI levels to differentiate between skeletal muscle damage versus cardiac muscle lesions (4, 7).

In daily practice there are demands for screening tests which are both sensitive and cost effective. Many studies have been published focusing on the cost effectiveness of testing for cTnI to rule out myocardial ischemia versus extended hospitalization of each patient suffering from angina. These studies concluded that the use of cTnI as a screening tool could significantly reduce the length of stay and average hospital costs (11). The authors found that there was a 25% decrease in total charges per patient admission and shorter length of stay when cTnI examination was used to rule out myocardial ischemia in patients with angina, indicating that testing for cTnI is an efficient and cost effective method (11, 13). Additionally, the percent of undiagnosed cardiac events was 1.1% when using cTnT to screen for a myocardial ischemia, but only 0.3% when cTnI was used as a marker and decreased hospital admissions by 11% (12). Furthermore, there were more deaths due to misdiagnosis when using cTnT than cTnI, and there were more false positive cTnT seen in patients with renal failure (12).

## CONCLUSION

Troponin T elevations, along with increase in CK-MB are used as markers of myocardial injury and have become part of our standard screening routine, but many conditions exist where elevated levels of these markers are falsely interpreted to indicate myocardial injury. For example, patients with inflammatory myositis (eg. dermatomyositis and polymyositis) have elevated levels of CK, CK-MB, sTnT and cTnT that arise due to the the continuous impairment and regeneration of skeletal muscle. The antibodies used to detect cTnT in the “Troponin T assay” cross-react with the various other subtypes of Troponin T, and elevated levels of these proteins are often misinterpreted as Troponin T (cTnT) (5). To delineate if the elevation in cardiac enzymes is due to inflammatory myositis or myocardial injury, a Troponin I (cTnI) test should be ordered (4, 7). With Troponin I levels we can decide if patients with myositis need further cardiac workup, or if the elevation in the enzymes is just associated to their disease.
